# Avoidance habit learning after recovery from anorexia nervosa: An fMRI study

**DOI:** 10.1111/jcpp.70204

**Published:** 2026-07-17

**Authors:** Clara M. Breier, Julius Hennig, Joseph A. King, Daniel Geisler, Arne Doose, Ilka Boehm, Maria Seidel, Tung Anh Nguyen, Katharina Zwosta, Veit Roessner, Uta Wolfensteller, Hannes Ruge, Stefan Ehrlich

**Affiliations:** ^1^ Translational Developmental Neuroscience Section, Division of Psychological and Social Medicine and Developmental Neurosciences, Faculty of Medicine TUD Dresden University of Technology Dresden Germany; ^2^ Faculty of Psychology TUD Dresden University of Technology Dresden Germany; ^3^ Department of General Psychology, Faculty of Psychology TUD Dresden University of Technology Dresden Germany; ^4^ Department of Child and Adolescent Psychiatry, Faculty of Medicine TUD Dresden University of Technology Dresden Germany

**Keywords:** anorexia nervosa, eating disorders, habits, self‐control, avoidance learning

## Abstract

**Background:**

Anorexia Nervosa (AN) has been linked to altered self‐regulatory control processes and maladaptive habitual avoidance behavior. Both goal‐directed and habit systems may be relevant to AN, in which restrictive eating behavior can appear highly deliberate and increasingly automatic over time. A previous fMRI study identified behavioral and neural markers hinting at heightened self‐regulatory control (‘overcontrol’) during an avoidance habit task in acutely underweight adolescents and young women with AN. Extending this research to recovered individuals with a history of AN (recAN), we aimed to clarify whether these findings persist and can be seen as trait vulnerabilities.

**Methods:**

A sample of 40 recAN and 40 pairwise age‐matched healthy control (HC) participants carried out a three‐phase experimental paradigm including (1) training of goal‐directed response‐outcome behavior, (2) training of avoidance stimulus–response pairings, and (3) a test of habitual response interference. Our fMRI analysis strategy focused on capturing training‐related changes in neural activity related to reinforcement learning processes.

**Results:**

Analyses of main effects of task confirmed expected behavioral and fMRI results. However, unlike previous findings of greater activation in frontoparietal control regions in young acutely underweight patients with AN during the establishment of avoidance behavior and of higher task performance, all behavioral task measures, region‐of‐interest fMRI, and follow‐up whole‐brain analyses yielded no group differences between recAN and HC.

**Conclusions:**

Our findings of intact avoidance habit learning after recovery from AN suggest that correlates of overcontrol previously observed in acutely underweight individuals with AN may reflect state‐dependent rather than persistent trait markers of the disorder.

## Introduction

Anorexia Nervosa (AN) is a severe psychiatric disorder characterized by extreme food restriction, disturbed body image, and fear of weight gain, which predominantly affects young women and typically emerges during adolescence (Mitchell & Peterson, 2020; Zipfel, Giel, Bulik, Hay, & Schmidt, 2015). AN has notably high chronicity, relapse, and mortality rates (Steinhausen, 2002); and its dysfunctional behaviors often persist beyond acute illness. Understanding the neurobiological mechanisms underlying these behaviors is essential for developing effective treatments (Bulik et al., 2022; Haynos, Anderson, Askew, Craske, & Peterson, 2021; Haynos, Widge, Anderson, & Redish, 2022; Kaye, Wierenga, Bailer, Simmons, & Bischoff‐Grethe, 2013; Keeler, Kan, Treasure, & Himmerich, 2024). Cognitive neuroscience accounts of goal‐directed and habitual control may be relevant for understanding the persistence of maladaptive behavior in AN.

Influential theories of AN attribute the development and persistence of AN to aberrant habit formation and to a psychological tendency sometimes referred to as ‘overcontrol’, characterized by heightened self‐regulation of behavior (e.g., Fairburn, Shafran, & Cooper, 1999; Schmidt & Treasure, 2006; Walsh, 2013). According to these complementary perspectives, maladaptive behaviors like restrictive eating and excessive exercise are initially reinforced by the subjective rewards of sustained effortful self‐regulation, including reduced anxiety, a sense of achievement, and perceived self‐worth. With repeated engagement, these behaviors may gradually shift from effortful, controlled goal‐directed actions toward habitual patterns that are increasingly automatic and resistant to change (Conceição et al., 2023; Seidel et al., 2022; Steinglass & Foerde, 2016; Walsh, 2013). Consequently, a fundamentally stronger (possibly domain‐general) propensity for habit formation may constitute a vulnerability factor for AN (Uniacke, Timothy Walsh, Foerde, & Steinglass, 2018; Walsh, 2013).

Most previous investigations of habit learning in AN have focused primarily on approach habits in appetitive (reward‐related) contexts, with mixed results. While some studies suggest enhanced habit formation in AN (Brown, Devine, Otto, Bischoff‐Grethe, & Wierenga, 2024; Favier et al., 2020), others report no difference to healthy control (HC) participants (Godier et al., 2016; Westwater et al., 2022; see also Foerde, 2025). Importantly, little is known about avoidance habit learning in AN, despite the central role of restrictive eating (i.e., food avoidance) in AN and reports that persisting avoidance (e.g., safety behaviors) might contribute to maintenance of the disorder (Melles, Spix, & Jansen, 2021; Spix & Jansen, 2025). We previously investigated behavioral and neural correlates of avoidance habit learning in acutely underweight adolescents and young women with AN (acAN; Hennig et al., 2025). While avoidance habit learning was not altered in acAN patients compared to HC, acAN patients exhibited superior behavioral performance (i.e., higher accuracies in the goal‐directed phases of a task, for both habit‐compatible and habit‐incompatible stimuli) and a stronger activation increase during successful habit learning in cognitive control‐associated brain regions, including the dorsomedial prefrontal cortex (dmPFC) and the inferior parietal lobule (IPL; Botvinick & Braver, 2015; Miller & Cohen, 2001). This pattern was largely consistent with clinical accounts of overcontrol in AN and suggested heightened engagement of self‐regulatory processes rather than altered habit learning per se. The absence of significant habit‐related effects may partly reflect the relatively short illness duration of a mostly adolescent cohort. In line with theoretical considerations (Walsh, 2013) and self‐report data (Davis, Walsh, Schebendach, Glasofer, & Steinglass, 2020), habit strength may increase over time with prolonged illness. Consequently, robust habit effects would more likely emerge in adults with a longer history of AN.

Interpreting neural alterations as trait‐like illness mechanisms in patients with AN is confounded by the effects of undernutrition on brain function and behavior (Phillipou, Rossell, & Castle, 2018). The acute state with low body mass index (BMI) is associated with reductions in gray matter volume and white matter integrity alongside metabolic, endocrine and behavioral changes (e.g., neuropsychological functioning) that, at least partially, normalize with weight gain during recovery (Fuglset, 2019; Miller, 2011; Scharner & Stengel, 2019). This raises the question of whether observed differences in neural activation and behavior (including performance in experimental psychological paradigms) reflect reversible, starvation‐related states of acute illness or predisposing traits contributing to vulnerability (Frank, 2013). As prospective longitudinal studies addressing this issue face practical challenges, such as low prevalence rates, examining individuals who are weight‐restored and have recovered from AN provides a valuable complementary approach to disentangle trait characteristics of AN from state‐dependent effects (Kaye, Fudge, & Paulus, 2009).

In the present fMRI study, our goals were twofold: First, we applied the avoidance habit learning paradigm from Hennig et al. (2025) to test for altered habit learning in individuals recovered from AN. Second, we extended this prior work to investigate whether the behavioral and neural patterns suggesting overcontrol in acAN patients persist in recovered participants. We hypothesized that elevated control and increased frontoparietal activity observed in acAN by Hennig et al. (2025) represent a trait (rather than state) marker of AN. Accordingly, we expected recovered patients to show similarly superior performance in goal‐directed task phases and elevated training‐related frontoparietal activity.

## Methods

### Sample

We analyzed data from 40 female recovered individuals with a history of AN (recAN) and 40 healthy controls (HC) after pairwise age‐matching and data quality control. An additional number of three participants (1 recAN, 2 HC) were excluded due to subpar behavioral performance (Appendix S1), and seven participants (3 recAN, 4 HC) were excluded after thorough quality control checks of the neuroimaging data. To preclude possible effects of developmental status between groups due to a larger number of younger participants in the HC sample (data from an additional 10 HC were collected for age requirements of a related study), we implemented a pairwise age‐matching algorithm (Munkres, 1957), resulting in the final sample. There was no sample overlap with the previous study by Hennig et al. (2025). Participants were recruited between 2017 and 2020 via advertisements among school and university students.

To be considered ‘recovered’, recAN participants had to have met the DSM‐5 AN criteria in the past, be menstruating, be maintaining a BMI ≥18.5 kg/m^2^ (if older than 18 years) or BMI >10th age percentile (if younger than 18 years) for at least 6 months prior to study participation, and have not engaged in significant restrictive eating patterns (or bingeing‐purging) during this time. On average, recAN had been weight‐recovered for 4.7 years (*SD* = 3.2), with 95% of participants weight‐recovered for 12+ months (sensitivity analyses excluding participants recovered <12 months are reported in Appendix S2 incl. Tables S1 and S2). HC participants had to have a current BMI of >10th age percentile or 18.5–28 kg/m^2^, be eumenorrheic and without history of psychiatric illness. Several exclusion criteria were applied to both groups including psychotropic medication within 4 weeks prior to the study and medical conditions that could affect appetite, eating behavior, or body weight (for further criteria, see Appendix S3).

The Institutional Review Board of the Technische Universität Dresden approved the study (EK 536122015) and all participants (and their guardians if underage) gave written informed consent.

### Clinical measures

All recAN participants had a past diagnosis of AN, which we confirmed using the Structured Interview for Anorexic and Bulimic Syndromes (SIAB‐EX, adapted to DSM‐5 criteria; Fichter & Quadflieg, 2001). The interview was also used to ascertain the absence of a current eating disorder in all participants. Additionally, we assessed participants for other psychiatric disorders via a structured clinical interview (MINI/MINI‐KID; Sheehan et al., 1998, 2010). Interviews were conducted by clinically experienced and trained research assistants under the supervision of a child and adolescent psychiatrist.

In addition to the clinical interviews, we administered questionnaires to characterize our sample and explore relationships with clinical variables. ED‐related symptoms were assessed with the Eating Disorders Inventory‐2 (EDI‐2; Paul & Thiel, 2005), general psychopathology with the Symptom Checklist‐90‐R (SCL‐90‐R; Franke & Derogatis, 2002), depressive symptoms with the Beck Depression Inventory‐II (BDI‐II; Hautzinger et al., 2009). BMI standard deviation scores were calculated for each participant to adjust for age and sex (BMI‐SDS; Kromeyer‐Hauschild et al., 2001). We used the Brief Self‐Control Scale (BSCS; Sproesser, Strohbach, Schupp, & Renner, 2011) to measure trait self‐control. We assessed sensitivity to punishment (and reward) with the Behavioral Inhibition/Activation System scales (BIS/BAS; Strobel, Beauducel, Debener, & Brocke, 2001) as a key motivational driver underlying avoidance behavior.

### Procedures and avoidance habit task

This study's paradigm was identical to that used in the previous study (Hennig et al., 2025; adapted from Zwosta, Ruge, Goschke, & Wolfensteller, 2018), implemented in E‐Prime 2.0 (Psychology Software Tools, Pittsburgh, PA). The habit task consisted of three experimental phases (see Figure 1 and Appendix S4 for a detailed task description). Phase 1 was performed outside of the MRI scanner, phases 2–3 inside. Phase 1 (132 trials) established goal‐directed behavior via hierarchical response‐outcome associations. Participants were cued to either ‘maintain’ or ‘switch’ the current frame color by responding to visual stimuli (three natural vs. three artificial pictograms). The stimulus category determined which button press (left or right) produced which color outcome. Phase 2 (392 trials) established avoidance habits through extensive overtraining. Participants learned stimulus–response (S‐R) associations between some of the previous pictograms (two of each category) and button presses via trial‐and‐error. They were punished for incorrect responses by losing points and thus part of their monetary compensation. In phase 3 (112 trials), habit strength was tested by reinstating the goal‐directed task from phase 1. Trials were either compatible to the phase 2 training (the required goal‐directed response matched the habitual avoidance response), incompatible (responses conflicted), or not associated with a trained habit (‘no habit’ condition). Because errors were no longer punished, persistence of the overtrained habitual button press in incompatible trials indicated habitual responding.

**Figure 1:**
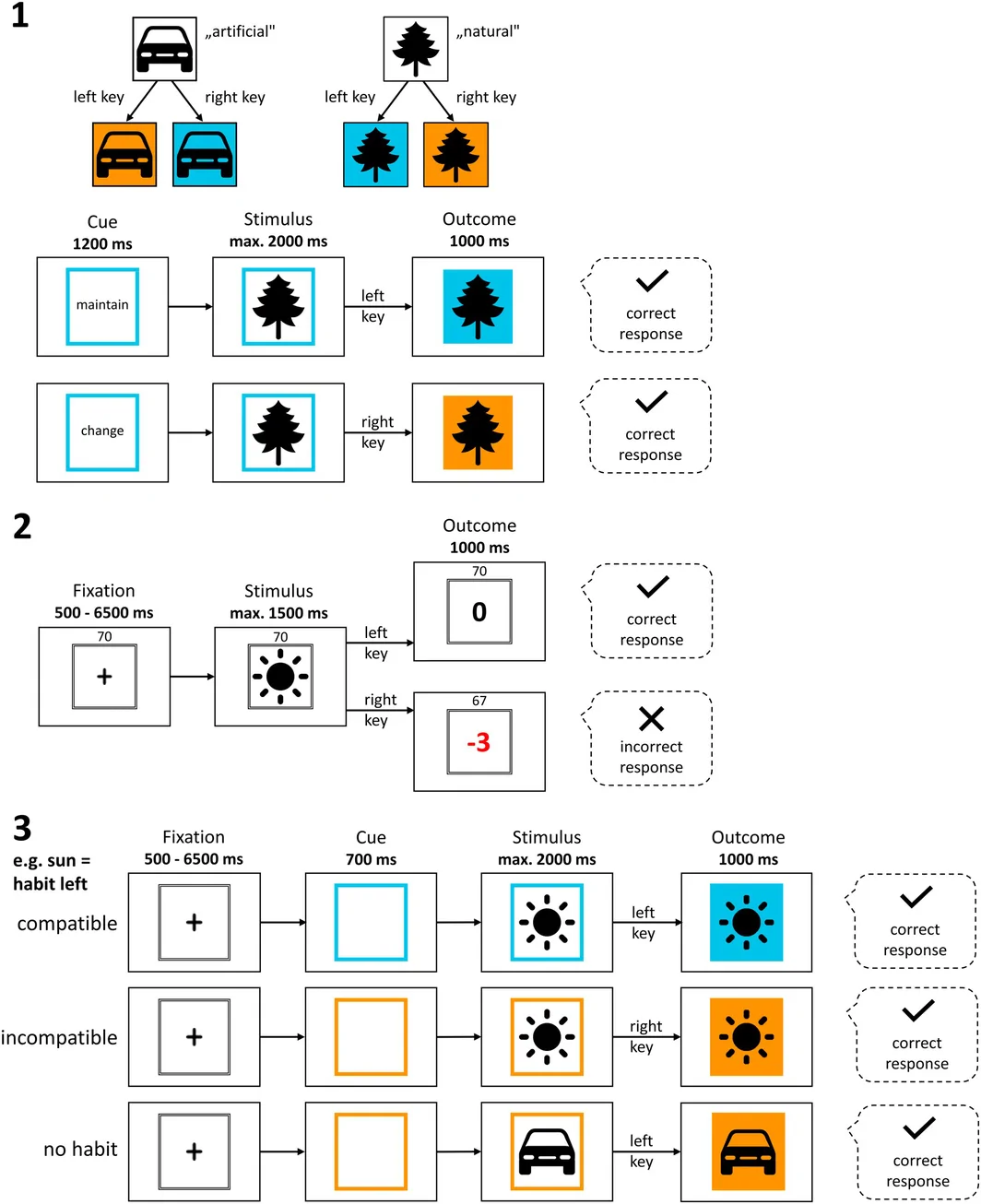
Experimental avoidance habit learning paradigm consisting of three consecutive phases. (1) Phase 1: Goal‐directed behavior was established by presenting hierarchical response‐outcome associations using two color cues (orange, blue) and two stimulus categories (3 ‘artificial’ and 3 ‘natural’ stimuli). Additionally, the trials consisted of a ‘maintain’ or ‘change’ cue in order to prevent habit learning. (2) Phase 2: Trials involving punishment were used to train habitual avoidance behavior through extensive training of stimulus–response–outcome associations, which had to be identified by trial and error. (3) Phase 3: The overtrained stimulus–response associations of phase 2 were put into competition with goal‐directed behavior to test individual habit strength using the contingencies learned in phase 1 (without the ‘maintain’ and ‘change’ cue). Trials could be compatible when the correct response based on the color cue and category matched the response learned in phase 2 for a certain stimulus and incompatible when they did not match. Additionally, ‘no habit’ trials were based on the stimuli that were not used in phase 2. For visualization purposes only, dotted speech bubbles indicate whether a depicted response was considered correct. Figure adapted from Hennig et al. (2025). ‘Avoidance habit learning in adolescents and young women with anorexia nervosa: An fMRI study’, https://doi.org/10.1111/jcpp.70019, used under CC BY 4.0 (http://creativecommons.org/licenses/by/4.0). This figure is licensed under CC BY 4.0 (http://creativecommons.org/licenses/by/4.0).

### Structural and functional image acquisition and data processing

Functional and structural images were acquired using sequences identical to Hennig et al. (2025) on a 3T Siemens TRIO MRI scanner (Erlangen, Germany; Appendix S5). Corresponding to Hennig et al. (2025), functional and structural images were processed with SPM12 (http://www.fil.ion.ucl.ac.uk/spm/software/spm12/) within the Nipype framework (Gorgolewski et al., 2011), including the use of simultaneous temporal slice‐timing and motion correction and a sample‐specific DARTEL template (for more details, see Appendix S6; Ashburner, 2007; Roche, 2011). We evaluated fMRI data quality by manual inspection using the artifact detection tool (http://www.nitrc.org/projects/artifact_detect). Volumes that exceeded an intensity threshold of three standard deviations or a threshold of 2 mm normalized movement in any direction were classified as outliers and excluded from statistical models (motion outlier: recAN: 0.22 ± 0.48; HC: 0.50 ± 1.04; intensity outlier: recAN: 6.95 ± 3.73; HC: 8.02 ± 3.08; both group comparisons n.s. with *p* > .13).

### Statistical analyses

Demographic, clinical, and behavioral task‐based data were analyzed using Python (version 3.11.9) and R (version 4.3.1). The behavioral metrics of interest were error rate (ER) and reaction times (RT) in all three phases (see Appendix S7 for details). To verify that the participants had begun to show goal‐directed responses after initial orientation with the task, we grouped the first attempt at each trial of phase 1 into four consecutive blocks, identical to Hennig et al. (2025). We conducted an analysis of variance (ANOVA) incorporating blocks and cue (maintain vs. switch) as within‐subject factors and group as between‐subject factor. This was done to examine potential learning effects, which would be indicated by a decrease in ER. For phase 2, we ran ANOVAs for ER and RT with training (block 1–7) as within‐subject factor and group as between‐subject factor to show successful acquisition of the S‐R contingencies. For phase 3, we computed ANOVAs for ER and RT in the goal‐directed trials with compatibility (compatible vs. incompatible) as within‐subject factor and group as between‐subject factor to validate our experimental approach. Behavioral analyses were family‐wise error (FWE) corrected via the Holm method. Additionally, as in Hennig et al. (2025), we computed a ‘habit‐driven compatibility effect’ metric for phase 3 for both RT and ER by subtracting the values of the incompatible condition from the compatible condition. Consequently, the more negative the value, the stronger the habit. We used this measure to further explore the associations between behavioral habit strength in phase 3 and clinical variables that either exhibited group differences or that are related to the theoretical constructs of interest.

Statistical models of the fMRI data were built consistent with Hennig et al. (2025), focusing on the training processes during phase 2 and their ability to predict phase 3 behavior. More specifically, at the single‐subject level, we employed an event‐related design in which stimulus onsets were modeled separately for trials with correct, incorrect, or omitted responses. Only correct trials included a parametric modulator reflecting the cumulative number of correct responses at each trial, designed to reflect BOLD response changes specific to successful ongoing S‐R avoidance habit learning (hereafter ‘training‐related changes’). All models additionally included six motion parameters and nuisance regressors for each motion and intensity outlier volume identified during preprocessing.

In an initial proof‐of‐principle analysis, we examined general effects of habit learning, consistent with Zwosta et al. (2018) and Hennig et al. (2025), by conducting whole‐brain analyses across all participants using the training regressor described above. After confirming the expected pattern of task‐related activation, we performed a whole‐brain voxel‐wise comparison to assess potential group differences in training‐related activity during avoidance habit learning.

To follow up on clusters showing group differences between acAN patients and HC identified by Hennig et al. (2025), we conducted a voxel‐wise analysis with a combined mask of regions extracted from Hennig et al. (2025), including dmPFC, IPL, posterior mid‐cingulate cortex (pMCC) and posterior Insula (pI). All analyses were FWE corrected using 3DClustSim with two‐sided thresholding and first‐nearest‐neighbor clustering (voxel‐wise *p* < .001, cluster‐wise *p* < .05; AFNI version 24.0.14).

Nonsignificant effects of group in the frequentist regions of interest (ROI) analyses or behavioral results in phase 3 were followed up with Bayesian *t*‐Tests and Bayesian repeated measures ANOVAs, respectively, to quantify evidence in favor of the null hypothesis. These tests were performed via JASP (JASP Team, 2024; Wagenmakers et al., 2018) using default priors (Cauchy distribution with *r* = .707 for *t*‐tests, multivariate Cauchy for ANOVAs; see Appendix S8 incl. Tables S3–S7 for details and robustness checks).

## Results

### Demographic and clinical measures

As expected, groups did not differ in age, BMI‐SDS, or IQ (Table 1), but the recAN group reported higher eating‐disorder‐related (EDI‐2), depressive (BDI‐II), and general psychological (SCL‐90‐R) symptom severity. RecAN participants also exhibited increased punishment sensitivity (BIS) compared to the HC group but showed no differences in self‐control (BSCS) or reward sensitivity (BAS).

**Table 1:** Demographic and clinical characteristics of the sample

	HC		recAN		*t*	*p*	*p*_FDR_	*d*
	*M*	*SD*	*M*	*SD*				
Age	22.32	3.83	22.38	3.72	−0.077	.939	.939	−0.02
Socioeconomic status	2.90	0.91	2.84	0.92	0.305	.761	.845	0.07
Time since recovery from AN (in years)	/	/	4.69	3.17				
BMI	21.46	1.91	21.31	1.87				
BMI‐SDS	−0.27	0.58	−0.31	0.66	0.306	.761	.845	0.07
IQ	112.20	12.33	116.32	10.99	−1.579	.118	.237	−0.35
EDI‐2 total	128.00	24.92	159.77	44.57	−3.886	**<.001**	.**003**	−0.88
SCL‐90‐R GSI	0.22	0.29	0.43	0.39	−2.759	.**007**	.**023**	−0.62
BDI‐II	4.15	4.79	9.01	9.09	−2.950	.**005**	.**023**	−0.67
BSCS total	47.00	7.18	46.31	6.20	0.452	.652	.841	0.10
BIS	20.72	3.28	22.72	3.44	−2.649	.**010**	.**024**	−0.60
BAS	40.73	5.12	39.75	4.76	0.881	.381	.519	0.20

			#	%				
Reported lifetime psychiatric disorders								
Depressive	/	/	14	35				
OCD	/	/	1	2.5				
Anxiety/panic	/	/	5	12.5				
PTSD	/	/	1	2.5				
AN subtype during acute illness								
Restrictive	/	/	26	64				
Binge‐purge	/	/	14	35				

Group differences were tested using two‐sided Welch's *t*‐tests, displaying mean and *SD*, *t* and *p* values (both unadjusted and FDR‐corrected). *P* values of significant group differences are marked in bold. As a measure of effect size, we included Cohen's *d*. Socioeconomic status was calculated based on academic skill level and international standard occupational classification (using a scale of 0–5; Appendix S3). The lower table section reports occurring psychiatric comorbidities in the recAN group. IQ = Intelligence Quotient based on a short version of the German adaption of the Wechsler Adult Intelligence Scale (von Aster et al., 2006). AN, Anorexia Nervosa; BAS, Behavioral Activation System; BDI‐II, Beck Depression Inventory‐II; BIS, Behavioral Inhibition System; BMI‐SDS, body mass index standard deviation score; BSCS, Brief Self‐Control Scale; EDI‐2, Eating Disorder Inventory‐2; HC, healthy control participants; OCD, obsessive‐compulsive disorder; PTSD, post‐traumatic stress disorder; recAN, recovered anorexia nervosa participants; SCL‐90‐R GSI, Symptom Checklist‐90‐R Global Severity Index.

### Behavioral results

#### Phase 1

Confirming that the first experimental phase was successful in eliciting goal‐directed behavior via hierarchical R‐O associations, marginal contrast analyses revealed that participants made fewer errors as blocks progressed during phase 1 of the experiment (all *p*s < .002 except block 2 vs. block 3, *p* = .085; after a main effect of block, *F*[3, 234] = 25.15, *p*_Holm_ < .001, *η*_p_^2^ = .244), and fewer errors in the maintain vs. the switch condition, *F*(1, 78) = 166.94, *p*_Holm_ < .001, *η*_p_^2^ = .682 (Figure 2). RT results showed a similar cue main effect, *F*(1, 78) = 178.36, *p*_Holm_ < .001, *η*_p_^2^ = .696; while the effect of block was not significant, *F*(2.47, 192.41) = 2.53, *p*_Holm_ = .423, *η*_p_^2^ = .031 (Greenhouse–Geisser corrected)—a pattern observed in our related work (Hennig et al., 2025). Importantly and in contrast to the group × block interaction in ER that was observed in our previous study and suggested steeper R‐O learning in acutely underweight patients with AN (Hennig et al., 2025), no group differences or interactions emerged here (*F*s < 1.35, *ps*_Holm_ = 1.0, *η*s_p_^2^ < .017).

**Figure 2:**
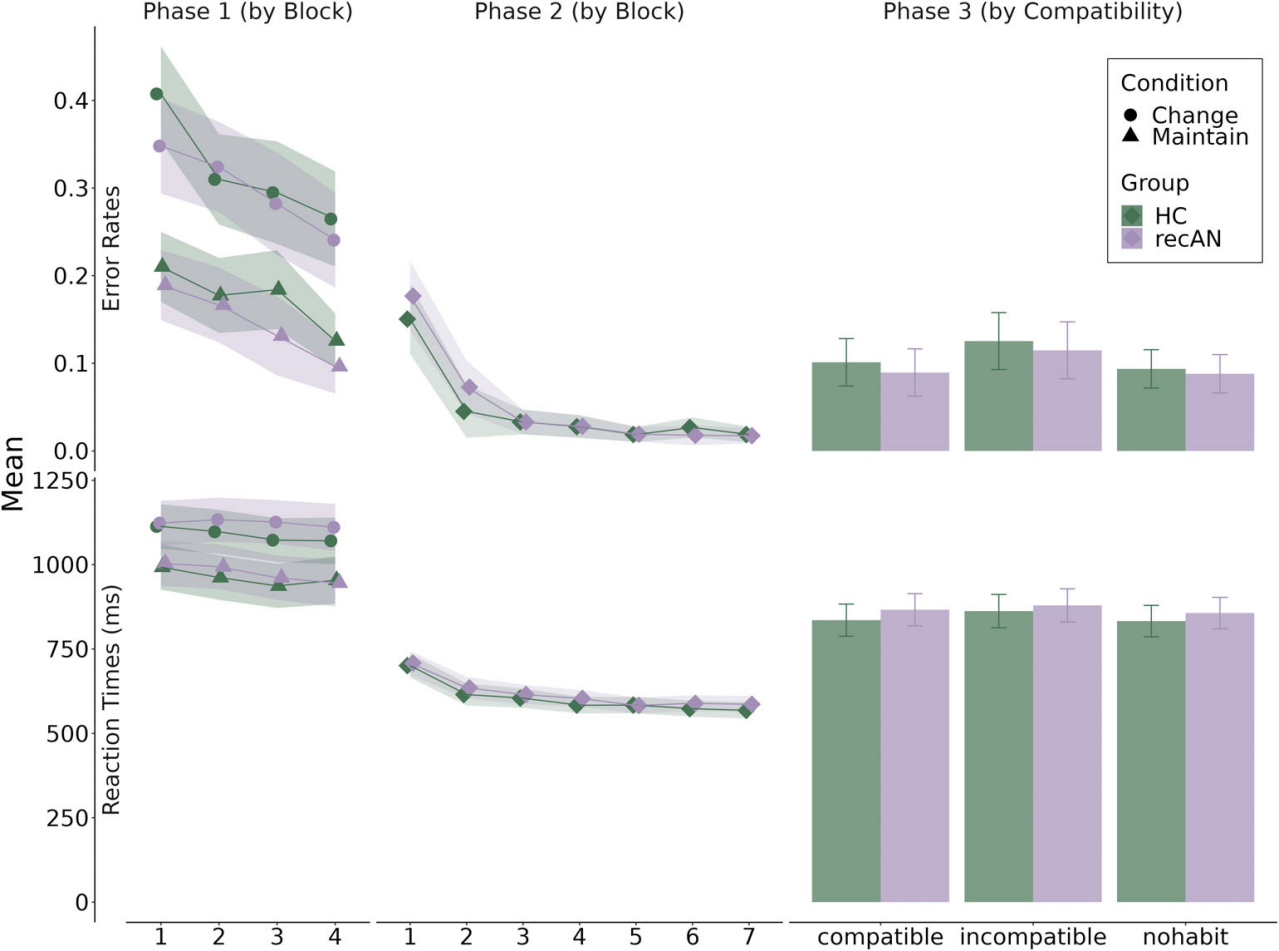
Behavioral results (error rates and reaction times) from the three experimental phases. The *Y* axis displays the mean performance in error rates (upper row) and reaction times (lower row, in ms) across all three experimental phases, separately for the (weight)‐recovered Anorexia Nervosa participants (recAN) and the healthy control (HC) group. The results for phases 1 and 2 are binned into consecutive blocks to depict performance change over time, while the results of phase 3 are displayed separately for the incompatible, compatible, and no habit condition. Error bars and confidence bands denote 95% confidence intervals. The figure visibly displays the decrease of error rates and/or reaction times in phases 1 and 2 as well as the similar task performance in both groups across all conditions in phase 3.

#### Phase 2

As expected, a main effect of training during phase 2 of the experiment was found for both ERs (*F*[1.96, 152.52] = 65.18, *p*_Holm_ < .001, *η*_p_^2^ = .455) and RTs (*F*[2.55, 198.57] = 81.52, *p*_Holm_ < .001, *η*_p_^2^ = .511) in that responses became more accurate and faster during the course of the experiment. This confirmed that the intended overtraining of S‐R associations was successful. However, as in Hennig et al. (2025), no effects involving group reached significance (*F*s < 0.62, *ps*_Holm_ > .583, *η*s_p_^2^ < .016), indicating intact S‐R learning in recAN.

#### Phase 3

In the third phase, which tested the acquired habitual response to certain stimuli against the goal‐directed response pattern, a main effect of compatibility was significant in both ERs (*F*[2, 156] = 6.50, *p*_Holm_ = .007, *η*_p_^2^ = .077) and RTs (*F*[2, 156] = 6.94, *p*_Holm_ = .004, *η*_p_^2^ = .082), indicating slower and more inaccurate reactions when habitually learned and goal‐directed responses were incompatible (Appendix S9). Again, in contrast to the previously found ER main effect of group in acAN patients (Hennig et al., 2025), no significant main effects of group or interactions emerged (*F*s < 0.53, *ps*_Holm_ > .939, *η*s_p_^2^ < .007), suggesting that the avoidance habit overtraining did not disproportionately affect the performance of the recAN group.

Post hoc Bayesian repeated measures ANOVAs on ER and RT comparing all possible predictor combinations revealed that the models only involving the main effect of compatibility had the highest posterior model probability (Appendix S8 incl. Tables S5 and S6).

### Associations between experimental results and clinical measures

Exploratory correlational analyses between the behavioral compatibility effects (compatible–incompatible condition; a measure of habit strength) in ER and RT during phase 3 in the recAN group with BMI‐SDS, EDI‐2 total, EDI‐2 perfectionism, SCL‐90‐R Global Severity Index, BIS, and BSCS scores revealed no significant relationships (all |*r*| < .168 and all *p*_Holm_ = 1.0).

### Neuroimaging results

A whole‐brain one‐sample *t*‐test confirmed training‐related activity changes in regions consistent with prior findings (Hennig et al., 2025; Zwosta et al., 2018; Appendix S10 incl. Figure S1, Table S8). However, no significant group differences between recAN and HC participants were found in either the whole‐brain or ROI analyses based on regions identified in Hennig et al. (2025; Appendix S11 incl. Figures S2 and S3; both FWE‐corrected with *p* < .05).

The post hoc Bayesian *t*‐tests on mean beta weights from training‐related ROIs (Hennig et al., 2025) showed either moderate (dmPFC, pMCC, IPL; BF_01_ ≥ 3.358) or anecdotal (pI; BF_01_ = 1.991) evidence against group difference between recAN and HC (Appendix S8 incl. Table S7). To examine links between training‐related BOLD activity changes (phase 2) and behavioral habit‐driven compatibility effects (phase 3), we calculated Spearman correlations between beta weights and the compatibility effects for ER and RT. None were significant (ER: |*r*| ≤ 0.12, *p* > .283; RT: |*r*| ≤ 0.13, *p* > .233).

### Exploratory comparisons with acute AN

Additional exploratory analyses including acAN patient data from Hennig et al. (2025) showed no significant differences between acutely ill and recovered participants in behavioral task measures. Neurally, the acAN group showed larger activation increases in the dmPFC and the IPL during phase 2 than recAN participants (Appendix S12 incl. Tables S9–S11).

## Discussion

The aim of the present study was to investigate potential alterations in avoidance habit learning among participants with a history of AN. Firstly, our results suggest that habit learning in an avoidance context appears intact in recAN (as previously observed in acAN; Hennig et al., 2025). Secondly, assuming that the improved performance and increased frontoparietal activation in acAN reflect heightened self‐regulatory control (Hennig et al., 2025), our results imply a relative reduction of this overcontrol after recovery. Specifically, we found no evidence for significant group differences between recAN and HC in behavior or fMRI activation (whole‐brain or ROI).

Behaviorally, no group differences reached significance across any of the three task phases. Importantly, the generally superior task performance and steeper decline of ER (i.e., increasing accuracy) previously observed in acAN patients (Hennig et al., 2025) was not evident in the current recAN sample. Neurally, while BOLD activity decreased during avoidance training progression in regions consistent with Zwosta et al. (2018) and Hennig et al. (2025), we observed no significant group differences between recAN and HC, even in frontoparietal control regions (dmPFC, IPL) previously identified in acAN patients. Together, these null findings point toward an apparent attenuation (‘normalization’) of overcontrol (in an avoidance context) after weight‐recovery from AN, suggesting that the previously observed alterations in acAN reflect state factors associated with acute undernutrition, rather than traits.

### Normalization of overcontrol in AN

The acute state of AN seems to be linked to overcontrol and extreme goal pursuit (Decker, Figner, & Steinglass, 2015; Foerde et al., 2021; Haynos et al., 2022; Kaye et al., 2013; King et al., 2025; Pauligk et al., 2025; Steinglass et al., 2012), which may impede cognitive flexibility in decision‐making (Buzzichelli, Marzola, Amianto, Fassino, & Abbate‐Daga, 2018; Schmidt & Treasure, 2006). However, these control‐related markers may be state‐dependent and not persist following recovery. Indeed, findings after recovery remain mixed: while some studies report persistent alterations (Bernardoni et al., 2021; Boehm et al., 2016; Holliday, Tchanturia, Landau, Collier, & Treasure, 2005; Kaufmann et al., 2023; King et al., 2019; Ritschel et al., 2017; Tenconi et al., 2010; Wierenga et al., 2015), others observe normalized cognitive functions and connectivity (Boehm et al., 2021; Bracké et al., 2025; Castro‐Fornieles et al., 2019; Doose et al., 2020; Fuglset, 2019; King et al., 2020; Sarrar et al., 2011; Tchanturia et al., 2007). Regarding the specific task used here, Zwosta et al. (2018) established that behavioral outcomes are primarily influenced by the goal‐directed control system. Consistent with this, the increased frontoparietal activity observed in acute AN during habit training suggests that this paradigm engages sustained cognitive control (Hennig et al., 2025). Notably, this overactivity was absent in our recovered sample. We speculate that continuous effortful self‐regulation (e.g., of eating, hunger and in general) may no longer be particularly necessary after recovery, resulting in a relative normalization of frontoparietal activity. Consequently, both behavioral performance and neural activation patterns in recAN did not differ significantly from HC. Interpreting our results as indicators of a normalization process is supported by exploratory direct comparisons finding lower task‐related dmPFC and IPL activation increase in recAN than in acAN. However, this explanation attempt warrants caution because these observed neural differences did not translate into significant behavioral differences. Further, as acAN and recAN varied in age, our additional analyses do not fully account for confounding developmental differences and maturation effects (Boehm, Mohr, & Ehrlich, 2022). Alternatively, less pronounced control might constitute a protective factor that facilitates recovery from AN. Future computational modeling (e.g., model‐based vs. model‐free control) could clarify whether behaviors observed in AN stem from shared goal‐directed and habitual control mechanisms or from an imbalance between these systems (Brown et al., 2024; Conceição et al., 2023; Foerde, 2025; Haynos et al., 2022; Radzikowska, Pike, & Hall‐McMaster, 2025; Rigoli & Martinelli, 2021; Wierenga, Brown, & Reilly, 2025).

Considering the centrality of restrictive eating in AN, we focused on avoidance learning as an important disorder maintenance mechanism (Foerde, 2025; Melles et al., 2021). In the context of value‐based decision‐making, individuals with AN tend to experience conflicting choices less strongly in situations requiring self‐control (Bernardoni et al., 2023; Fürtjes et al., 2022; King et al., 2016). Interestingly, this has also been observed in recovered individuals (Bernardoni et al., 2025), which may reflect a trait toward effortless or habitual self‐control. In fact, the processing of reward and the perception of the salience of rewarding/punishing cues are likely altered in AN and warrant further study (Frank, 2013; Murray, Brown, Kaye, & Wierenga, 2022; O'Hara, Campbell, & Schmidt, 2015; Rye, Amorim, Ward, & Milton, 2025). However, applying these findings to avoidance behavior requires caution, because approach and avoidance behaviors seem to differ in terms of mechanisms and complexity (LeDoux & Daw, 2018).

In contrast to acAN, where neural activity in the pMCC and pI did not decrease during training (Hennig et al., 2025), activity in these areas in our recAN group did not differ from HC. This could be interpreted in light of their suspected role in processing aversive stimuli and reactivity to punishment (Hayes & Northoff, 2012; Kim, Yoon, Kim, & Hamann, 2015; Murao et al., 2017; Wade‐Bohleber et al., 2021), which is often elevated in AN (Harrison, O'Brien, Lopez, & Treasure, 2010; Jappe et al., 2011; King et al., 2022). Although neural and behavioral indicators of heightened punishment sensitivity seem to normalize with recovery, self‐reported punishment sensitivity remains increased (Abber, Murray, Brown, & Wierenga, 2024; Frank, Collier, Shott, & O'Reilly, 2016; Jonker, Glashouwer, & De Jong, 2022). The same was the case in our recAN sample (Table 1), suggestive of persistently elevated behavioral inhibition. However, the effect size (*d* = 0.60) was notably smaller than in the acAN group (*d* = 1.09).

### What do we learn about habits?

Our lack of significant group differences in both the experimental avoidance habit measure and activation in brain regions generally associated with habit formation is in line with two studies in AN (Godier et al., 2016; Westwater et al., 2022), but not with another study using a task that arbitrates between model‐based and model‐free control, which also has been linked to habits (Foerde et al., 2021). One possible explanation for these null findings is the relatively short illness duration in our sample (*M* = 3.1, *SD* = 1.9 years), which is likely linked to participants' young age (*M* = 22.4, *SD* = 3.7 years). While this relative youth is advantageous, as it yields in a more homogeneous sample with few comorbidities, it limits our ability to examine associations between illness duration and habit strength (exploratory correlations between illness duration and ROI betas or habit strength were nonsignificant, |*r*s| < .18, *p*s > .359). Further studies on samples with heterogeneous illness histories are needed to clarify how illness duration may interact with disorder‐specific or domain‐general habit development in AN, and how this might relate to the ability to sustain recovery. However, recruitment of such recovered participants remains a major challenge.

Importantly, age itself may also shape habit‐related processes: Prior work has shown differences in activity of habit‐related neural regions during food choice tasks between adolescents and adults with AN (Foerde, Steinglass, Shohamy, & Walsh, 2015; Lloyd et al., 2025; see also Foerde, 2025). In this context, the absence of behavioral differences in our young adult sample may possibly also reflect ongoing neurodevelopmental maturation, for example of frontoparietal control networks, beyond effects attributable to weight restoration alone.

Further, pronounced habitual avoidance in AN might emerge most strongly in real‐life contexts (Seidel et al., 2022, 2024) compared to lab settings, especially when using disorder‐unrelated stimuli instead of food (for recent evidence on the context‐dependence of goal‐directed/habitual control, see Wagner, Frölich, Schwöbel, Smolka, & Kiebel, 2026).

## Limitations

A number of limitations, especially regarding our task and sample, should be noted. Reliably inducing habits and distinguishing them from goal‐directed behavior remains a challenge in human experimental research (De Wit et al., 2018; Gera et al., 2023; Hardwick, Forrence, Krakauer, & Haith, 2019; Pool et al., 2022; Watson & De Wit, 2018). While our task succeeded in eliciting compatibility effects in both ER and RT during phase 3, small effect sizes and overall low ERs indicate that the task was quite easy. As task demands seem to play a role in modulating the inhibitory control‐related PFC response in recAN (Oberndorfer, Kaye, Simmons, Strigo, & Matthews, 2011), perhaps a more demanding task would have allowed for the detection of subtler group differences. Additionally, the task has not yet been applied to real‐life settings to test naturalistic habit formation. Furthermore, while our study examines avoidance habit learning on one hand and how neural and behavioral changes in AN (Hennig et al., 2025) might normalize after recovery on the other hand, our task did not directly assess cognitive/inhibitory control processes.

Separating effects of emaciation from premorbid vulnerability factors of AN remains crucial but challenging. As prospective studies are difficult to implement, studying recovered patients is a valuable approach in addition to more complex studies like twin designs (Seidel et al., 2020). However, the definition of ‘recovery’ from AN remains the subject of discussion (Bardone‐Cone, Hunt, & Watson, 2018; De Rijk et al., 2024). In our study, while criteria for recovery included the return of menstruation and largely normalized eating behavior, recAN participants still reported elevated eating disorder‐related and general psychological symptoms. In addition, possible scarring processes should be considered when interpreting the present findings.

Following suggestions that individuals with the binge‐purge subtype of AN might be more susceptible to punishment (Glashouwer, Bloot, Veenstra, Franken, & De Jong, 2014; Murao et al., 2017), which drives avoidance behavior, further studies of subtype differences in avoidance habit learning might be promising.

Lastly, due to our entirely female sample, we concur that our results cannot necessarily be generalized to boys or young men with AN.

## Conclusion

Like previously observed in acutely underweight AN (Hennig et al., 2025), the current study found intact avoidance habit learning in individuals recovered from AN. However, and in contrast to Hennig et al., recovered individuals showed no significantly altered behavioral or neural markers of overcontrol during the avoidance habit task. These results suggest that avoidance‐related behavioral and neural correlates of heightened self‐regulatory control may normalize following (weight‐)recovery from AN. They might therefore reflect a consequence of acute undernutrition rather than a trait factor or an enduring ‘scar’ of AN. Future research using different tasks should investigate the potential roles of illness severity and chronicity. Nevertheless, the present findings offer a hopeful outlook for patients in that clinical interventions specifically targeting aspects of overcontrol (e.g., DBT, Lynch et al., 2013; ACT, Onnink, Konstantinidou, Moskovich, Karekla, & Merwin, 2022) might be helpful in treating AN.

## Ethical considerations

The Institutional Review Board of the Technische Universität Dresden (IRB00001473) approved the study (Reference number: EK 536122015, approved March 1, 2016) and all participants (and their guardians if underage) gave written informed consent.

**Key points**

**What is known?**

- Anorexia nervosa (AN) may be associated with heightened self‐regulatory control (‘overcontrol’) and altered habit learning.
- Little is known about avoidance habit learning in AN, but persisting avoidance (e.g., safety behaviors) might contribute to disorder maintenance.
- Prior fMRI research in acutely underweight adolescents and young women with AN showed evidence of overcontrol during avoidance habit learning.

**What is new?**

- This follow‐up study in individuals recovered from AN found no behavioral or neural differences compared to healthy controls, suggesting both intact avoidance habit learning and that the previously observed overcontrol markers are state‐related rather than traits.

**What is relevant?**

- These findings highlight that maladaptive control processes can normalize with recovery. Clinically, interventions targeting overcontrol may be helpful.

## Supporting information


**Appendix S1.** Quality control of behavioral data.
**Appendix S2.** Sensitivity analysis without *recAN* participants recovered for <12 months.
**Table S1.** Demographic and clinical characteristics of the sample without recAN participants recovered for < 12 months.
**Table S2.** Repeated‐measures ANOVA of the behavioral data of Phases 1, 2 and 3 of the sample without recAN participants recovered for <12 months.
**Appendix S3.** Data collection and measures.
**Appendix S4.** Experimental procedure (additional information).
**Appendix S5.** Structural and functional image acquisition.
**Appendix S6.** Functional image data processing and analysis.
**Appendix S7.** Treatment of RT outliers.
**Appendix S8.** Post‐hoc Bayesian statistics.
**Table S3.** Model comparison: Bayesian repeated measures ANOVA of Phase 3 error rates.
**Table S4.** Model comparison: Bayesian repeated measures ANOVA of Phase 3 reaction times.
**Table S5.** Model comparison: Bayesian repeated measures ANOVA of Phase 3 error rates for two alternative prior specifications.
**Table S6.** Model comparison: Phase 3 reaction times bayesian repeated measures ANOVA for two alternative prior specifications.
**Table S7.** Results of the Bayesian independent samples *t*‐Tests (recAN vs. HC).
**Appendix S9.** Behavioral data, Phase 3.
**Appendix S10.** Neuroimaging results: general task effects.
**Figure S1.** Whole‐brain fMRI results of the Phase 2 main effect of successful S‐R training.
**Table S8.** Whole‐brain results of the Phase 2 main effect of successful S‐R training. FWE‐corrected at *p* < .05, voxel‐wise *p* < .001.
**Appendix S11.** Neuroimaging results: group contrasts.
**Figure S2.** ROI fMRI results of the Phase 2 main effect of successful S‐R training (recAN > HC).
**Figure S3.** Whole‐brain fMRI results of the Phase 2 main effect of successful S‐R training (recAN > HC).
**Appendix S12:** Exploratory comparisons between ACAN, RECAN, HC.
**Table S9.** Demographic and clinical comparison between acutely ill patients and recovered individuals.
**Table S10.** Repeated‐measures ANCOVA of the behavioral data of Phases 1, 2 and 3 with acutely ill patients, recovered individuals and healthy controls.
**Table S11.** Results of univariate analyses of covariance comparing ROI beta values between acutely ill patients, recovered individuals and healthy controls.

## Data Availability

The data that support the findings of this study are available on request from the corresponding author. The data are not publicly available due to privacy or ethical restrictions.
