# Supplementary material for: Avoidance habit learning after recovery from anorexia nervosa: An fMRI study
Source: J Child Psychol Psychiatry. 2026 Jul 17;67(12):2056–68. doi: 10.1111/jcpp.70204 (PMC13745786; doi:10.1111/jcpp.70204)
Supplement: Supplementary file 1 [file JCPP-67-2056-s001.docx]

**Avoidance Habit Learning after Recovery from Anorexia Nervosa: An fMRI Study**

***Supporting Information***

[Appendix S1. Quality Control of Behavioral Data	2](#_Toc233966440)

[Appendix S2: Sensitivity Analysis without recAN participants recovered for <12 months	2](#_Toc233966441)

[Appendix S3. Data Collection & Measures	5](#_Toc233966442)

[Appendix S4. Experimental Procedure (Additional Information)	6](#_Toc233966443)

[Appendix S5. Structural and Functional Image Acquisition	8](#_Toc233966444)

[Appendix S6. Functional Image Data Processing and Analysis	8](#_Toc233966445)

[Appendix S7. Treatment of RT Outliers	9](#_Toc233966446)

[Appendix S8. Post-Hoc Bayesian Statistics	9](#_Toc233966447)

[Appendix S9. Behavioral Data, Phase 3	14](#_Toc233966448)

[Appendix S10. Neuroimaging Results: General Task Effects	14](#_Toc233966449)

[Appendix S11. Neuroimaging Results: Group Contrasts	18](#_Toc233966450)

[Appendix S12: Exploratory comparisons between acAN, recAN, HC	20](#_Toc233966451)

[References	24](#_Toc233966452)

## Appendix S1. Quality Control of Behavioral Data

For behavioral measures, quality control criteria were (a) an accuracy of ≥85% in Phase 2 and (b) ≥60% of Phase 3 of the habit induction experiment, following Hennig et al. (2025). These performance limits were introduced to ensure that participants understood the task and the instructions and were able to perform above guessing probability.

## Appendix S2: Sensitivity Analysis without recAN participants recovered for <12 months

Excluding the two recAN participants who were recovered for <12 months did not change the significance of the results, as evident below (Table S1 for demographics, Table S2 for behavioral ANOVAs).

In addition, correlations between the phase 3 compatibility effects in ER and RT) with clinical variables BMI-SDS, EDI-2 total, SCL-90-R Global Severity Index, BIS and BSCS scores in the 12+ months recovered recAN group remained non-significant (all |*r*|<.17 and all *p*_Holm_=1.0).

Likewise, correlations between the phase 3 compatibility effects and mean beta weights of the training-related activity changes during phase 2 remained not significant in the sample without the two recAN participants who were recovered for <12 months (all |*r*|<.14 and all *p*>.24).

**Table S1**

*Demographic and Clinical Characteristics of the Sample Without recAN Participants Recovered for <12 Months*

|  | HC | | recAN | |  |  |  |  |
| --- | --- | --- | --- | --- | --- | --- | --- | --- |
|  | *M* | *SD* | *M* | *SD* | *t* | *p* | *p*_FDR_ | *d* |
| Age | 22.32 | 3.83 | 22.14 | 3.65 | 0.213 | .832 | .863 | -0.05 |
| Socioeconomic status | 2.90 | 0.91 | 2.84 | 0.92 | 0.305 | .761 | .845 | 0.07 |
| Time since recovery from AN (in years) | / | / | 4.69 | 3.17 |  |  |  |  |
| BMI | 21.46 | 1.91 | 21.34 | 1.89 |  |  |  |  |
| BMI-SDS | -0.27 | 0.58 | -0.29 | 0.66 | 0.173 | .863 | .863 | 0.04 |
| IQ | 112.20 | 12.33 | 117.08 | 10.72 | -1.868 | .066 | .131 | -0.42 |
| EDI-2 total | 128.00 | 24.92 | 157.82 | 44.09 | -3.597 | **<.001** | **.007** | -0.83 |
| SCL-90-R GSI | 0.22 | 0.29 | 0.43 | 0.40 | -2.663 | **.010** | **.032** | -0.61 |
| BDI-II | 4.15 | 4.79 | 8.71 | 9.03 | -2.726 | **.009** | **.032** | -0.63 |
| BSCS total | 47.00 | 7.18 | 46.27 | 6.12 | 0.474 | 0.637 | .863 | 0.11 |
| BIS | 20.72 | 3.28 | 22.63 | 3.51 | -2.467 | **.016** | **.040** | -0.56 |
| BAS | 40.73 | 5.12 | 39.79 | 4.77 | 0.835 | .406 | .677 | 0.19 |

*Note.* Group differences were tested using two-sided Welch’s t-tests, displaying mean and SD, *t* and *p* values (both unadjusted and FDR-corrected). *P* values of significant group differences are marked in bold. As a measure of effect size, we included Cohen’s *d*. Socioeconomic status was calculated based on academic still level and international standard occupational classification (using a scale of 0-5; Appendix S3). *N*=38 recAN, 40 HC. Of the 38 recAN participants, *n*=16 reported other (lifetime) psychiatric disorders (*n*=13 depressive disorders, *n*=1 obsessive-compulsive disorder, *n*=4 anxiety disorders, *n*=1 post-traumatic stress disorder, *n*=1 panic disorder). *N*=25 recAN participants had been of the restrictive subtype and *n*=13 of the binge-purge subtype during the time of acute illness. IQ=Intelligence Quotient based on a short version of the German adaption of the Wechsler Adult Intelligence Scale (von Aster et al., 2006). AN, Anorexia Nervosa; BAS, Behavioral Activation System; BDI‐II, Beck Depression Inventory‐II; BIS, Behavioral Inhibition System; BMI‐SDS, body mass index standard deviation score; BSCS, Brief Self‐Control Scale; EDI‐2, Eating Disorder Inventory‐2; HC, healthy control participants; OCD, obsessive‐compulsive disorder; PTSD, post‐traumatic stress disorder; recAN, recovered anorexia nervosa participants; SCL‐90‐R GSI, Symptom Checklist‐90‐R Global Severity Index.

**Table S2**

*Repeated-Measures ANOVA of the Behavioral Data of Phases 1, 2 and 3 of the Sample Without recAN Participants Recovered for <12 Months*

| Predictor | Error Rates | | | | | | Reaction Times | | | | |  |
| --- | --- | --- | --- | --- | --- | --- | --- | --- | --- | --- | --- | --- |
|  | *df* | *F* | *p* | *p*_Holm_ | *η*²_partial_ | *df* | | *F* | *p* | *p*_Holm_ | *η*²_partial_ | |
| *Phase 1* | | | | | | | | | | | | |
| cue | 1, 76 | 162.06 | **<.001** | **<.001** | .681 | 1, 76 | | 169.84 | **<.001** | **<.001** | .691 | |
| block | 3, 228 | 25.32 | **<.001** | **<.001** | .250 | 2.48, 188.57 ^a^ | | 2.44 | .077 | .423 | .031 | |
| group | 1, 76 | 0.98 | .326 | 1.0 | .013 | 1, 76 | | 0.22 | .642 | 1.0 | .003 | |
| cue × block | 2.67, 203.01 ^a^ | 1.25 | .292 | 1.0 | .016 | 3, 228 | | 0.57 | .634 | 1.0 | .007 | |
| cue × group | 1, 76 | 0.15 | .696 | 1.0 | .002 | 1, 76 | | 0.44 | .511 | 1.0 | .006 | |
| block × group | 3, 228 | 0.84 | .475 | 1.0 | .011 | 2.48, 188.57 ^a^ | | 0.41 | .709 | 1.0 | .005 | |
| cue × block × group | 2.67, 203.01 ^a^ | 0.94 | .416 | 1.0 | .012 | 3, 228 | | 0.70 | .551 | 1.0 | .009 | |
| *Phase 2* | | | | | | | | | | | | |
| training | 1.94, 147.25 ^a^ | 60.77 | **<.001** | **<.001** | .444 | 2.47, 188.01 ^a^ | | 77.87 | **<.001** | **<.001** | .506 | |
| group | 1, 76 | 0.51 | .477 | .583 | .007 | 1, 76 | | 0.32 | .571 | .918 | .004 | |
| training × group | 1.94, 147.25 ^a^ | 1.12 | .327 | .583 | .015 | 2.47, 188.01 ^a^ | | 0.60 | .586 | .918 | .008 | |
| *Phase 3* | | | | | | | | | | | | |
| compatibility | 2, 156 | 6.50 | **.002** | **.007** | .077 | 2, 156 | | 6.94 | **.001** | **.004** | .082 | |
| group | 1, 78 | 0.31 | .580 | 1.0 | .004 | 1, 78 | | 0.53 | .470 | .939 | .007 | |
| compatibility × group | 2, 156 | 0.07 | .933 | 1.0 | <.001 | 2, 156 | | 0.46 | .631 | .939 | .006 | |

*Note. N*=38 recAN, 40 HC. *p*-values are reported both uncorrected and Holm-corrected for multiple testing. *P* values of significant group differences are marked in bold. As a measure of effect size, we included partial *η*².

^a^ = Greenhouse-Geisser-corrected

## Appendix S3. Data Collection & Measures

Further clinical data were collected from all participants using a semi-structured research interview. This in-house interview has been used continuously in our ongoing studies on AN (e.g., Doose et al., 2020; Ehrlich et al., 2015; King et al., 2016; Seidel et al., 2024) in addition to the SIAB-EX (Fichter & Quadflieg, 2001) and MINI/MINI-KID interviews (Sheehan et al., 1998, 2010). The goal of this procedure is a comprehensive description of our participant groups. Using the semi-structured interview, trained interviewers assess demographic and clinical data in the following domains: Socio-economic status (e.g., educational level, occupation, current living situation), ethnicity, family psychiatric history, detailed information on body weight, including highest and lowest lifetime body mass index (BMI) and weight changes over the past six weeks, detailed information on menstrual cycle and contraceptives intake, current and past medical history, current and past psychiatric or psychological treatments, current and past medication intake and nicotine abuse.

IQ was assessed with a short version of the German adaption of the Wechsler Adult Intelligence Scale (von Aster et al., 2006). The socioeconomic status was calculated based on household occupational status and international standard occupational classification (Ganzeboom et al., 1992), and highest educational skill level (0-5). Higher values thus indicate a higher socioeconomic status. Inter-rater reliability of the interviews we used are 0.81 for current and 0.85 for past diagnoses measured by the SIAB-EX (Fichter & Quadflieg, 2001) and between 0.76 and 0.93 for the Mini-International Neuropsychiatric Interview (Sheehan et al., 1998).

Additional exclusion criteria applied to both groups included consumption of any psychotropic substance within the past 4 weeks (except for SSRIs in recAN; *n*=4 in the current sample), any history of organic brain syndrome, schizophrenia, substance dependence, psychosis not otherwise specified (NOS), or bipolar disorder; current inflammatory, neurologic, or metabolic disorders (psychiatric diagnoses were based on DSM-IV). Further, participants were not included if they were <12 or ≥30 years old, were currently excessively overweight (BMI >28 if older than 18 years or BMI >94^th^ age percentile if younger than 18 years), were pregnant or breast feeding, had anemia, or if their estimated IQ was <85. HC were further excluded if they had a lifetime BMI below the tenth age percentile (if <18 years) or below 17.5 kg/m² (if >18 years).

Study data were collected and managed using the secure, web-based electronic data capture tool REDCap (Research Electronic Data Capture; Harris et al., 2009).

## Appendix S4. Experimental Procedure (Additional Information)

The habit task (Zwosta et al., 2018) employed in this study consisted of three consecutive phases. Immediately prior to performing each phase, participants received standardized written instructions explaining the rules. The mappings of the stimuli categories were counterbalanced. Identical to Hennig et al. (2025), we modified the original task in the following ways: (1) of the original two conditions “reward” and “punishment” in phase 2, we only used the punishment condition to focus on avoidance learning; (2) we reduced the number of stimuli (three artificial and three natural) and consequentially the total number of trials in each phase; and (3) we replaced stimuli associated with food (mushroom, cow) to prevent an adverse reaction by the AN group. To this end, we used stimuli depicting a sun, a tree and a flower for the natural category and a scissor, a car and a ball for the artificial category.

Phase 1: Establishing goal-directed behavior (132 trials). This phase was intended to establish goal-directed behavior using hierarchical response-outcome (R-O) associations. Participants were instructed to maintain or switch the outcome color depending on the stimulus category (artificial or natural with three stimuli each), which was associated with a left or right button press. In detail, each trial started with a “maintain” or “switch” cue, framed by a blue or orange colored square. This cue was then replaced by one of the six stimuli, and participants then pressed a button to switch or maintain the current outcome color based on the stimulus category. The stimulus background then changed to the respective outcome color. The word “Error” was displayed below the square for incorrect responses, and the trial was repeated until the response was correct. Responses were either a left or a right index finger response on the buttons “d” and “k” on a QWERTZ laptop keyboard. Phase 1 also had a practice phase with the instruction consisting of 12 trials, during which participants could ask clarifying questions. The present color of a cue was always the outcome color that was produced in the previous trial, or a random color in the first trial. The R‑O contingencies were preserved even in error trials. For example, if the task was to maintain a blue color but the incorrect response was made, an orange outcome color would appear instead.

Phase 2: Avoidance habit learning (392 trials). This phase aimed to establish avoidance habits by overtraining certain stimulus-response (S-R) associations as participants tried to avoid punishment of incorrect responses. Negative feedback was given as deductions from total earnings over seven task blocks of 56 trials each. Two of the three stimuli from both categories were reused, with a randomly selected 2×2 set, each presented in 98 trials. At the beginning, participants were informed that the categories were now irrelevant and that they had to determine the correct left or right response for each of the four stimuli through trial-and-error. For both categories, one stimulus was associated with a right and one with a left response. Each trial commenced with a fixation cross within a black-and-white lined square, followed by the presentation of a stimulus. Participants received immediate feedback on their point outcome after responding. The total number of points accumulated during the current task block was displayed at the top of the screen. Participants started each block with 70 points, losing 3 points for each incorrect response while correct responses did not change the point total. Each of the 7×70 points equaled €0.01, allowing the participants to gain a maximum bonus of €4.90 (additionally to a €25 base rate compensation for their participation). Negative outcomes (“-3” points) were presented in red and neutral outcomes (“0” points) in black.

Phase 3: Testing habit strength/slip-of-action stage (112 trials). This phase was intended to test the habit-driven compatibility effect by putting the goal-directed behavior established in phase 1 (without the “switch” and “maintain” cues) into competition with the habitual avoidance behavior trained in phase 2. Participants were informed that they would no longer lose points, removing the S-R-O contingency. In detail, each trial began with a fixation cross, followed by a colored frame (blue or orange) and one of the six stimuli at the center of the frame. Based on the R-O contingencies of phase 1, trials were either habit-compatible or habit-incompatible.

## Appendix S5. Structural and Functional Image Acquisition

In accordance with guidelines for brain imaging investigations in eating disorder populations (Frank et al., 2018), we acquired all MRI data between 8 and 9 AM following an overnight fast to control for acute nutritional intake and diurnal hormone rhythms.

Images were obtained using a 3T Siemens TRIO MRI scanner equipped with a 32-channel head coil. The T1-weighted structural brain scans were acquired with a rapid acquisition gradient echo (MP-RAGE) sequence: number of slices=176; repetition time (TR)=1900 ms; echo time (TE)=2.26 ms; flip angle (FA)=9°; slice thickness=1 mm; voxel size=isotropic 1 mm; field of view (FoV)=256 mm x 224 mm; bandwidth=200 Hz/pixel).

Functional images were acquired using a gradient-echo T2*-weighted echo planar imaging (EPI) sequence with parameters as following: tilted 17° from anterior/posterior commissure (AC-PC) line toward coronal (to reduce signal dropout in orbitofrontal regions); number of volumes=435; number of slices=36; TR=2070 ms; TE=25 ms; FA=80°; in-plane resolution=3 mm; slice thickness=3.2 mm (resulting in a voxel size of 3 mm x 3 mm x 3.2 mm); FoV=192 mm ×192 mm; bandwidth=2232 Hz/pixel.

During fMRI, stimuli were presented using the E-Prime 2.0 software (Psychology Software Tools, Pittsburgh, PA).

## Appendix S6. Functional Image Data Processing and Analysis

Functional and structural images were processed with SPM12 (http://www.fil.ion.ucl.ac.uk/spm/software/spm12/) within the Nipype framework (http://nipy.sourceforge.net/nipype/; Gorgolewski et al., 2011). The functional images were corrected for temporal slice-timing and motion simultaneously using realign4D (Roche, 2011). The six realignment parameters, characterizing the rigid-body movement (x, y, z, pitch, roll, yaw), were saved and later used as nuisance covariates to account for the variance due to motion. Subsequently these images were coregistered to the participant’s structural brain image. A DARTEL template was created using structural images from all participants (Ashburner, 2007). The EPI volumes were then normalized to Montreal Neurological Institute space using the DARTEL template and corresponding flow field. The resulting data were smoothed with an isotropic 8 mm full-width at half-maximum Gaussian kernel and physiological noise was subtracted with CompCor (Behzadi et al., 2007). During the image data processing at the single participant-level using a general linear model (GLM), all regressors were convolved with a synthetic hemodynamic response function as implemented in SPM12.

## Appendix S7. Treatment of RT Outliers

For analysis of the behavioral response time (RT) data, only RTs of correctly answered trials were processed further. To minimize possible bias in the distribution of reaction times due to outlier exclusion, RTs were treated according to the transformation method outlined in Berger and Kiefer (2021). For each participant, block and experimental condition (where applicable), we therefore transformed the RT distribution into a symmetrical shape, *z*-standardized it and excluded all RTs below the cut-off value of -2*z* from the data set. Since a maximum RT of 2000 ms was predetermined by the experimental setup, we did not trim the upper end of the RT distribution this way. All subsequent calculations were then performed on the untransformed RTs.

## Appendix S8. Post-Hoc Bayesian Statistics

For the Bayesian repeated measures ANOVAs on the behavioral ER and RT data in Phase 3 of the experiment (with compatibility—compatible vs. incompatible—as within-subject factor and group as between-subject factor), we used the default uniform model priors in JASP (coefficient priors: fixed effects=0.5, random effects=1, covariates=0.354).

In ER, the model including only compatibility was the best-performing model (Table S3). In general, there was moderate to very strong evidence against models including any main effect or interaction of group (all BF_01_ ≥ 3.006). An analysis comparing models containing the effect of group with models without this effect (see Van Den Bergh et al., 2023; Wagenmakers et al., 2018) also indicated moderate evidence for excluding group (BF_excl_=3.005; this means that aggregated across all models, the posterior odds for excluding the factor group are approximately three times as large as the prior odds) and strong evidence for excluding the group×compatibility interaction (BF_excl_=11.828).

Similarly, in RT, the model only containing compatibility performed best (Table S4). The data provide anecdotal evidence against the model including main effects of group and compatibility (BF_01_=1.627; that is, the observed data are approximately 1.6 times more likely under the best performing model only containing compatibility than under the model that also has the main effect of group), while all other models performed substantially worse (all BF_01_ ≥ 13.808). Across all models, there was anecdotal evidence for excluding the main effect of group (BF_excl_=1.632), and moderate evidence for excluding the group×compatibility interaction (BF_excl_=8.486).

In order to check robustness of the results below, we reanalyzed the data using two different additional prior specifications, one wider (i.e., Cauchy r=1 for fixed effects) and one narrower (i.e., Cauchy r=0.2 for fixed effects). Results for these analyses did not change substantially, that is, the model including only compatibility remained the best-performing model in all cases (Tables S5, S6), and evidence for excluding group as a main effect or interaction ranged between anecdotal to moderate with the narrow Cauchy priors (ER: BFs_excl_ 1.700–3.519, RT: BFs_excl_ 1.211–2.993) and between anecdotal to very strong with the wider priors (ER: BFs_exclu_ 5.239–39.502, RT: BFs_excl_ 2.372–26.311).

Table S7 presents the Bayes Factors for the independent samples *t­*-tests performed on the group differences in mean beta values extracted from all four regions of interest. The Bayes Factor robustness checks implemented in JASP indicated that for all *t*-tests, evidence for the null hypothesis robustly remained in the anecdotal to moderate range for a broad range of Cauchy prior width specifications.

**Table S3**

*Model Comparison: Bayesian Repeated Measures ANOVA of Phase 3 Error Rates.*

| Models | P(M) | P(M\|data) | BF_M_ | BF_01_ | Error % |
| --- | --- | --- | --- | --- | --- |
| compatibility | 0.200 | 0.686 | 8.720 | 1.000 |  |
| compatibility + group | 0.200 | 0.228 | 1.182 | 3.006 | 1.450 |
| Null model (incl. subject and random slopes) | 0.200 | 0.050 | 0.212 | 13.623 | 1.083 |
| compatibility + group + compatibility * group | 0.200 | 0.019 | 0.079 | 35.555 | 2.709 |
| Group | 0.200 | 0.017 | 0.068 | 40.812 | 1.381 |

*Note.* All models include subject, and random slopes for all repeated measures factors. Models are compared with the best-performing model. P(M)=prior probability of the model, P(M|data)=posterior probability of the model, BF_M_=change of model odds from prior to posterior, BF_01_=Bayes Factor of the model comparison (the best-performing model is compared to each other model), Error%=numerical precision of the result.

**Table S4**

*Model Comparison: Bayesian Repeated Measures ANOVA of Phase 3 Reaction Times.*

| Models | P(M) | P(M\|data) | BF_M_ | BF_01_ | Error % |
| --- | --- | --- | --- | --- | --- |
| compatibility | 0.200 | 0.565 | 5.185 | 1.000 |  |
| compatibility + group | 0.200 | 0.347 | 2.125 | 1.627 | 4.679 |
| compatibility + group + compatibility * group | 0.200 | 0.041 | 0.171 | 13.808 | 7.242 |
| Null model (incl. subject and random slopes) | 0.200 | 0.030 | 0.124 | 18.706 | 0.641 |
| Group | 0.200 | 0.017 | 0.071 | 32.330 | 3.656 |

*Note.*  All models include subject, and random slopes for all repeated measures factors. Models are compared with the best-performing model. P(M)=prior probability of the model, P(M|data)=posterior probability of the model, BF_M_=change of model odds from prior to posterior, BF_01_=Bayes Factor of the model comparison (the best-performing model is compared to each other model), Error%=numerical precision of the result.

**Table S5**

*Model Comparison: Bayesian Repeated Measures ANOVA of Phase 3 Error Rates for Two Alternative Prior Specifications.*

| Models | P(M) | P(M\|data) | BF_M_ | BF_01_ | Error % |
| --- | --- | --- | --- | --- | --- |
| *Cauchy prior width r=1* | | | | | |
| compatibility | 0.200 | 0.727 | 10.629 | 1.000 |  |
| compatibility + group | 0.200 | 0.125 | 0.573 | 5.795 | 2.880 |
| Null model (incl. subject and random slopes) | 0.200 | 0.122 | 0.558 | 5.933 | 2.664 |
| group | 0.200 | 0.022 | 0.092 | 32.334 | 3.120 |
| compatibility + group + compatibility * group | 0.200 | 0.003 | 0.013 | 232.320 | 3.001 |
| *Cauchy prior width r=0.2* | | | | | |
| compatibility | 0.200 | 0.541 | 4.714 | 1.000 |  |
| compatibility + group | 0.200 | 0.324 | 1.916 | 1.670 | 2.745 |
| compatibility + group + compatibility * group | 0.200 | 0.091 | 0.399 | 5.960 | 3.643 |
| Null model (incl. subject and random slopes) | 0.200 | 0.028 | 0.117 | 18.988 | 0.761 |
| group | 0.200 | 0.016 | 0.065 | 34.061 | 2.400 |

*Note.* All models include subject, and random slopes for all repeated measures factors. Models are compared with the best-performing model. P(M)=prior probability of the model, P(M|data)=posterior probability of the model, BF_M_=change of model odds from prior to posterior, BF_01=_Bayes Factor of the model comparison (the best-performing model is compared to each other model), Error%=numerical precision of the result.

**Table S6**

*Model Comparison: Phase 3 Reaction Times Bayesian Repeated Measures ANOVA for Two Alternative Prior Specifications.*

| Models | P(M) | P(M\|data) | BF_M_ | BF_01_ | Error % |
| --- | --- | --- | --- | --- | --- |
| *Cauchy prior width r=1* | | | | | |
| compatibility | 0.200 | 0.620 | 6.514 | 1.000 |  |
| compatibility + group | 0.200 | 0.263 | 1.426 | 2.357 | 5.690 |
| Null model (incl. subject and random slopes) | 0.200 | 0.077 | 0.333 | 8.063 | 0.926 |
| group | 0.200 | 0.031 | 0.127 | 20.145 | 3.829 |
| compatibility + group + compatibility * group | 0.200 | 0.010 | 0.040 | 62.015 | 8.681 |
| *Cauchy prior width r=0.2* | | | | | |
| compatibility | 0.200 | 0.467 | 3.510 | 1.000 |  |
| compatibility + group | 0.200 | 0.371 | 2.363 | 1.259 | 4.015 |
| compatibility + group + compatibility * group | 0.200 | 0.128 | 0.585 | 3.661 | 5.216 |
| Null model (incl. subject and random slopes) | 0.200 | 0.019 | 0.075 | 25.233 | 0.493 |
| group | 0.200 | 0.015 | 0.061 | 31.022 | 3.340 |

*Note.* All models include subject, and random slopes for all repeated measures factors. Models are compared with the best-performing model. P(M), prior probability of the model; P(M|data), posterior probability of the model; BF_M_, change of model odds from prior to posterior; BF_01_, Bayes Factor of the model comparison (the best-performing model is compared to each other model); Error%=numerical precision of the result.

**Table S7**

*Results of the Bayesian Independent Samples* t*-Tests (recAN vs. HC).*

| ROI | BF_01_ | Error % | Posterior distribution of effect size δ | | |
| --- | --- | --- | --- | --- | --- |
|  |  |  | Median | 95% CI lower | 95% CI upper |
| pMCC | 4.183 | 0.017 | -0.049 | -0.458 | 0.357 |
| dmPFC | 3.767 | 0.017 | -0.106 | -0.519 | 0.299 |
| IPL | 3.358 | 0.016 | -0.145 | -0.560 | 0.261 |
| pI | 1.991 | 0.014 | -0.258 | -0.682 | 0.150 |

*Note.* The estimated Bayes factor BF_01_ indicates the ratio of the likelihood of the data in favor of the null hypothesis compared to the two-sided alternative hypothesis. δ,standardized effect size, population version of Cohen’s *d;* dmPFC, dorsomedial prefrontal cortex; Error%, numerical precision of the result; HC, healthy control participants; IPL, inferior parietal lobe; pI, posterior insula; pMCC, posterior midcingulate cortex; recAN, recovered anorexia nervosa participants; SE, standard error.

## Appendix S9. Behavioral Data, Phase 3

Post-hoc marginal contrast analysis of the group×compatibility ANOVAs in Phase 3 revealed that participants in both groups had increased error rates (ER) and longer RTs in the incompatible than in the compatible condition (ER: Δ=‑0.02, 95%-CI [-0.05; -0.00], *t*=‑2.58, *p*=.023; RT: Δ=-20, 95%-CI [-39; -1], *t*=-2.59, *p*=.023) and compared to the condition without habitually trained responses (ER: Δ=0.03, 95%-CI [0.01; 0.05], *t*=3.38, *p*=.003; RT: Δ=26, 95%‑CI [9; 43], *t*=3.73, *p*=.001). There were no significant differences between the no-habit and the compatible conditions, *t*s<0.86, *p*s>.391.

## Appendix S10. Neuroimaging Results: General Task Effects

The initial whole-brain analysis across all participants revealed general activity patterns of habit training that were similar to Hennig et al. (2025) and Zwosta et al. (2018). Specifically, there was relative BOLD signal increase in bilateral temporal areas (including the left middle and superior temporal gyrus, left temporal pole, right superior temporal gyrus), the left supramarginal gyrus, left posterior orbital gyrus and gyrus rectus (FWE-corrected *p*<.05; see Figure S1, Table S8). Further, a relative training-related signal decrease was present in a broad range of regions including lateral frontal and parietal regions, medial frontal regions, the basal ganglia, the bilateral insula, the fusiform gyrus, the thalamus, bilateral occipito-temporal regions and the right middle frontal gyrus.

**Figure S1**

*Whole-brain fMRI Results of the Phase 2 Main Effect of Successful S-R Training.*

*Note*. Depicted are brain regions showing activity increase (red) and decrease (blue) related to the parametric modulator of the cumulative number of correct responses at each trial during phase 2 across all participants, thus capturing activity changes linearly related to successful S-R training. *T*-map, FWE-corrected at *p*<.05 via 3DClustStim. The contoured regions depict voxels surviving statistical thresholding, while surrounding activation that does not reach the analysis threshold is depicted translucently (Allen et al., 2012; Taylor et al., 2023).

**Table S8**

*Whole-Brain Results of the Phase 2 Main Effect of Successful S-R Training. FWE-corrected at p<.05, voxel-wise p<.001.*

| direction | region of peak voxel | peak voxel MNI coordinates (x, y, z) | | | *T*_max_ | cluster size |
| --- | --- | --- | --- | --- | --- | --- |
| decrease | Inferior Occipital gyrus L | -33 | -78 | -12 | 10.24 | 10501 |
|  | Precentral gyrus L | -36 | -9 | 58 | 9.47 |  |
|  | Postcentral gyrus L | -48 | -24 | 50 | 9.01 |  |
|  | Cerebellum R | 21 | -66 | -54 | 4.81 | 116 |
|  | Cerebellum L | -33 | -72 | -50 | 5.02 | 73 |
|  | Precuneus R | 9 | -66 | 50 | 4.19 | 30 |
| increase | Superior temporal gyrus R | 57 | -6 | -0 | 5.32 | 171 |
|  | Superior temporal gyrus R | 63 | -18 | 4 | 4.20 |  |
|  | Middle temporal gyrus L | -63 | -9 | -8 | 4.89 | 134 |
|  | Superior temporal gyrus L | -51 | -3 | -8 | 4.20 |  |
|  | Heschl’s gyrus L | -39 | -27 | 8 | 3.91 |  |
|  | White Matter/undefined | -30 | -45 | 4 | 5.52 | 102 |
|  | Temporal pole/middle temporal gyrus L | -51 | 12 | -31 | 5.42 | 61 |
|  | Supramarginal gyrus L/Angular Gyrus L/ White Matter | -36 | -57 | 27 | 4.81 | 49 |
|  | Gyrus rectus L | -0 | 39 | -19 | 4.07 | 44 |
|  | Gyrus Rectus L | -3 | 51 | -15 | 3.66 |  |
|  | Posterior orbital gyrus L | -42 | 30 | -15 | 4.46 | 36 |

*Note.* MNI coordinates=Montreal Neurological Institute coordinates, *T*_max_=peak intensity *t* value. Positive *T*_max_ values represent a training-related linear increase of activity, negative *T*_max_ represent a decrease. Regions were determined via the Automatic Anatomical Labeling Atlas (AAL3v1; Rolls et al., 2020) integrated in xjview (https://www.alivelearn.net/xjview).

## Appendix S11. Neuroimaging Results: Group Contrasts

**Figure S2**

*ROI fMRI Results of the Phase 2 Main Effect of Successful S-R Training (recAN > HC).*

*Note.* (A) T-Map of the region-of-interest (ROI) based training-related group contrast recAN > HC. A combined mask of all four ROIs was used (pMCC, posterior midcingulate cortex; dmPFC, dorsomedial prefrontal cortex; IPL, inferior parietal lobe; pI, posterior insula). Contours show the location of ROIs. No activation difference survived voxel-wise correction at p<.001 and cluster-wise FWE‑correction at p<.05, but subthreshold activation differences are depicted translucently for context (Allen et al., 2012; Taylor et al., 2023). (B) Violin plot of the mean beta values derived from training-related regression slopes during experimental phase 2 in all ROIs (PFC, IPL, pMCC, pI). The horizontal line denotes the group mean. recAN=recovered anorexia nervosa participants, HC=healthy control participants.

**Figure S3**

*Whole-Brain fMRI Results of the Phase 2 Main Effect of Successful S-R Training (recAN > HC).*

*Note.* Depicted are brain regions showing activity increase (red) and decrease (blue) related to the parametric modulator of the cumulative number of correct responses at each trial during phase 2 across the group contrast recAN > HC. T-map, FWE-corrected at p<.05 via 3DClustStim. The contoured regions depict voxels surviving statistical thresholding (importantly, this is not applicable here due to no cluster surviving FWE correction), while surrounding activation that does not reach the analysis threshold is depicted translucently (Allen et al., 2012; Taylor et al., 2023). recAN=recovered anorexia nervosa participants, HC=healthy control participants.

## Appendix S12: Exploratory comparisons between acAN, recAN, HC

To compare demographic, behavioral and neuroimaging responses between acAN, recAN and HC groups within one statistical model, we conducted additional group comparisons of demographic and questionnaire variables, exploratory repeated-measure ANCOVAs of phase 1, 2 and 3 ER and RT results and on extracted ROI beta values (from pMCC, dmPFC, IPL and pI). All comparisons were performed identically to the analyses reported in the main manuscript, but additionally included age as a covariate due to the recAN group being systematically older than the acAN group. For results, see Tables S9, S10 and S11 below. Behaviorally, no differences between the acAN and the recAN groups emerged. In the neuroimaging data, acAN participants showed a training-related BOLD activity increase (during experimental phase 2) in the dmPFC and IPL compared to not only HC, but also recAN participants. These results should be interpreted with caution as acAN and recAN are not matched on age and thus, possible confounding effects of age and developmental status cannot be ruled out.

**Table S9**

*Demographic and Clinical Comparison Between Acutely Ill Patients and Recovered Individuals.*

|  | acAN | | recAN | |  |  |  |  |
| --- | --- | --- | --- | --- | --- | --- | --- | --- |
|  | *M* | *SD* | *M* | *SD* | *t* | *p* | *p*_FDR_ | *d* |
| Age | 16.14 | 2.14 | 22.38 | 3.72 | -9.32 | <.001 | <.001 | -2.06 |
| Socioeconomic status | 3.60 | 0.84 | 2.84 | 0.92 | -18.21 | <.001 | <.001 | 0.87 |
| Parental socioeconomic status | 3.27 | 0.72 | 3.54 | 0.76 | -1.67 | .098 | .161 | -0.36 |
| Time since recovery from AN (in years) | / | / | 4.69 | 3.17 |  |  |  |  |
| BMI | 14.48 | 1.55 | 21.31 | 1.87 |  |  |  |  |
| BMI-SDS | -3.41 | 1.19 | -0.31 | 0.66 | -15.10 | <.001 | <.001 | -3.23 |
| EDI-2 total | 218.74 | 49.22 | 159.77 | 44.57 | 5.65 | <.001 | <.001 | 1.25 |
| SCL-90-R GSI | 1.15 | 0.66 | 0.43 | 0.39 | 6.05 | <.001 | <.001 | 1.32 |
| BDI-II | 25.52 | 10.63 | 9.01 | 9.09 | 7.63 | <.001 | <.001 | 1.67 |
| BSCS total | 44.21 | 8.39 | 46.31 | 6.20 | -1.22 | .227 | .309 | -0.27 |
| BIS | 22.77 | 3.08 | 22.72 | 3.44 | 0.06 | .955 | .955 | 0.01 |
| BAS | 40.57 | 3.82 | 39.75 | 4.76 | 1.02 | .310 | .388 | 0.23 |

*Note.* Group differences were tested using two-sided Welch’s t-tests, displaying mean and SD, *t* and *p* values (both unadjusted and FDR-corrected). *P* values of significant group differences are marked in bold. As a measure of effect size, we included Cohen’s *d*. *N*=40 recAN, 45 acAN participants. Socioeconomic status was calculated based on academic still level and international standard occupational classification (using a scale of 0-5; Appendix S3). IQ was not included in this analysis due to acAN and recAN group receiving partly different test editions. AcAN, acutely ill anorexia nervosa participants; AN, Anorexia Nervosa; BAS, Behavioral Activation System; BDI‐II, Beck Depression Inventory‐II; BIS, Behavioral Inhibition System; BMI‐SDS, body mass index standard deviation score; BSCS, Brief Self‐Control Scale; EDI‐2, Eating Disorder Inventory‐2; OCD, obsessive‐compulsive disorder; PTSD, post‐traumatic stress disorder; recAN, recovered anorexia nervosa participants; SCL‐90‐R GSI, Symptom Checklist‐90‐R Global Severity Index.

**Table S10**

*Repeated-Measures ANCOVA of the Behavioral Data of Phases 1, 2 and 3 With Acutely Ill Patients, Recovered Individuals and Healthy Controls.*

| Predictor | Error Rates | | | | | | Reaction Times | | | | |  |
| --- | --- | --- | --- | --- | --- | --- | --- | --- | --- | --- | --- | --- |
|  | *df* | *F* | *p* | *p*_Holm_ | *η*²_partial_ | *df* | | *F* | *p* | *p*_Holm_ | *η*²_partial_ | |
| *Phase 1* | | | | | | | | | | | | |
| cue | 1, 166 | 380.27 | **<.001** | **<.001** | .696 | 1, 166 | | 335.17 | **<.001** | **<.001** | .669 | |
| block | 3, 498 | 41.20 | **<.001** | **<.001** | .199 | 2.56, 425.22 ^a^ | | 2.30 | .087 | .869 | .014 | |
| group | 2, 166 | 2.04 | .134 | .802 | .024 | 2, 166 | | 0.10 | .906 | 1.00 | .001 | |
| age | 1, 166 | 6.44 | **.012** | .109 | .037 | 1, 166 | | 0.03 | .869 | 1.00 | <.001 | |
| cue × block | 2.85, 473.22 ^a^ | 2.46 | .065 | .520 | .015 | 3, 498 | | 1.15 | .327 | .520 | .007 | |
| cue × group | 2, 166 | 0.03 | .975 | 1.00 | <.001 | 2, 166 | | 1.01 | .365 | 1.00 | .012 | |
| cue × age | 1, 166 | 2.80 | .096 | .673 | .017 | 1, 166 | | 0.00 | .975 | 1.00 | <.001 | |
| block × group | 6, 498 | 1.42 | .204 | 1.00 | .017 | 5.12, 425.22 ^a^ | | 0.40 | .851 | 1.00 | .005 | |
| block × age | 3, 498 | 0.57 | .638 | 1.00 | .003 | 2.56, 425.22 ^a^ | | 0.71 | .525 | 1.00 | .004 | |
| cue × block × group | 5.70, 473.22 ^a^ | 0.56 | .756 | 1.00 | .007 | 6, 498 | | 0.34 | .916 | 1.00 | .004 | |
| cue × block × age | 2.85, 473.22 ^a^ | 0.54 | .648 | 1.00 | .003 | 3, 498 | | 0.58 | .625 | 1.00 | .004 | |
| *Phase 2* | | | | | | | | | | | | |
| training | 1.92, 318.71 ^a^ | 156.49 | **<.001** | **<.001** | .485 | 2.46, 408.25 ^a^ | | 114.58 | **<.001** | **<.001** | .408 | |
| group | 2, 166 | 0.39 | .678 | .718 | .005 | 2, 166 | | 0.31 | .730 | 1.00 | .004 | |
| age | 1, 166 | 3.63 | .058 | .233 | .021 | 1, 166 | | 0.73 | .394 | 1.00 | .004 | |
| training × group | 3.84, 318.71 ^a^ | 1.87 | .118 | .354 | .022 | 4.92, 408.25 ^a^ | | 0.79 | .555 | 1.00 | .009 | |
| training × age | 1.92, 318.71 ^a^ | 1.02 | .359 | .718 | .006 | 2.46, 408.25 ^a^ | | 3.58 | **.021** | .083 | .021 | |
| *Phase 3* | | | | | | | | | | | | |
| compatibility | 1.82, 302.34 ^a^ | 7.37 | **.001** | **.006** | .042 | 2, 332 | | 24.13 | **<.001** | **<.001** | .127 | |
| group | 2, 166 | 2.21 | .113 | .339 | .026 | 2, 166 | | 0.34 | .716 | 1.00 | .004 | |
| age | 1, 166 | 9.94 | **.002** | **.008** | .056 | 1, 166 | | 0.15 | .699 | 1.00 | <.001 | |
| compatibility × group | 3.64, 302.34 ^a^ | 0.90 | .456 | .913 | .011 | 4, 332 | | 0.92 | .454 | 1.00 | .011 | |
| compatibility × age | 1.82, 302.34 ^a^ | 0.49 | .596 | .913 | .003 | 2, 332 | | 0.77 | .464 | 1.00 | .005 | |

*Note.* Analyses included 45 acAN, 40 recAN and 85 HC participants. AcAN, acutely ill anorexia nervosa participants; HC, healthy control participants; recAN, recovered anorexia nervosa participants. *P* values are reported both uncorrected and Holm-corrected for multiple testing. *P* values of significant group differences are marked in bold. As a measure of effect size, we included partial *η*².

^a^ = Greenhouse-Geisser-corrected

**Table S11**

*Results of Univariate Analyses of Covariance Comparing ROI Beta Values Between Acutely Ill Patients, Recovered Individuals and Healthy Controls.*

| ROI | acAN  marginal mean (SE) | recAN  marginal mean (SE) | HC  marginal mean (SE) | *F* | *η*_p_² | Pairwise marginal contrasts | | |
| --- | --- | --- | --- | --- | --- | --- | --- | --- |
| pMCC | -0.0005 (0.0003) | -0.0012 (0.0003) | -0.0017 (0.0002) | 5.62 | .063 | acAN > HC (*p*=.004) | n.s. (*p*=.266) | n.s. (*p*=.266) |
| dmPFC | 0.0013 (0.0004) | -0.0001 (0.0003) | -0.0001 (0.0002) | 8.13 | .089 | acAN > HC (*p*<.001) | n.s. (*p*=.918) | acAN > recAN (*p*=.007) |
| IPL | 0.0031 (0.0005) | -0.0001 (0.0005) | 0.0002 (0.0003) | 14.05 | .145 | acAN > HC (*p*<.001) | n.s. (*p*=.616) | acAN > recAN (*p*<.001) |
| pI | 0.0002 (0.0004) | -0.0006 (0.0004) | -0.0008 (0.0003) | 1.92 | .023 | n.s. (*p*=.155) | n.s. (*p*=.694) | n.s. (*p*=.475) |

*Note.* Analyses included 45 acAN, 40 recAN and 85 HC participants. *F-*Tests for pMCC, dmPFC and IPL were significant. All comparisons included age as a covariate. Pairwise marginal contrasts were Holm-corrected for multiple testing. AcAN, acutely ill anorexia nervosa participants; dmPFC, dorsomedial prefrontal cortex; HC, healthy control participants; IPL, inferior parietal lobe; pI, posterior insula; pMCC, posterior midcingulate cortex; recAN, recovered anorexia nervosa participants; SE, standard error.

## References

Allen, E. A., Erhardt, E. B., & Calhoun, V. D. (2012). Data Visualization in the Neurosciences: Overcoming the Curse of Dimensionality. *Neuron*, *74*(4), 603–608. https://doi.org/10.1016/j.neuron.2012.05.001

Ashburner, J. (2007). A fast diffeomorphic image registration algorithm. *NeuroImage*, *38*(1), 95–113. https://doi.org/10.1016/j.neuroimage.2007.07.007

Behzadi, Y., Restom, K., Liau, J., & Liu, T. T. (2007). A component based noise correction method (CompCor) for BOLD and perfusion based fMRI. *NeuroImage*, *37*(1), 90–101. https://doi.org/10.1016/j.neuroimage.2007.04.042

Berger, A., & Kiefer, M. (2021). Comparison of Different Response Time Outlier Exclusion Methods: A Simulation Study. *Frontiers in Psychology*, *12*, 675558. https://doi.org/10.3389/fpsyg.2021.675558

Doose, A., King, J. A., Bernardoni, F., Geisler, D., Hellerhoff, I., Weinert, T., Roessner, V., Smolka, M. N., & Ehrlich, S. (2020). Strengthened Default Mode Network Activation During Delay Discounting in Adolescents with Anorexia Nervosa After Partial Weight Restoration: A Longitudinal fMRI Study. *Journal of Clinical Medicine*, *9*(4), 900. https://doi.org/10.3390/jcm9040900

Ehrlich, S., Geisler, D., Ritschel, F., King, J. A., Seidel, M., Boehm, I., Breier, M., Clas, S., Weiss, J., Marxen, M., Smolka, M. N., Roessner, V., & Kroemer, N. B. (2015). Elevated cognitive control over reward processing in recovered female patients with anorexia nervosa. *Journal of Psychiatry and Neuroscience*, *40*(5), 307–315. https://doi.org/10.1503/jpn.140249

Fichter, M. M., & Quadflieg, N. (2001). The structured interview for anorexic and bulimic disorders for DSM-IV and ICD-10 (SIAB-EX): Reliability and validity. *European Psychiatry*, *16*(1), 38–48. https://doi.org/10.1016/S0924-9338(00)00534-4

Frank, G. K. W., Favaro, A., Marsh, R., Ehrlich, S., & Lawson, E. A. (2018). Toward valid and reliable brain imaging results in eating disorders. *International Journal of Eating Disorders*, *51*(3), 250–261. https://doi.org/10.1002/eat.22829

Ganzeboom, H. B. G., De Graaf, P. M., & Treiman, D. J. (1992). A standard international socio-economic index of occupational status. *Social Science Research*, *21*(1), 1–56. https://doi.org/10.1016/0049-089X(92)90017-B

Gorgolewski, K., Burns, C. D., Madison, C., Clark, D., Halchenko, Y. O., Waskom, M. L., & Ghosh, S. S. (2011). Nipype: A Flexible, Lightweight and Extensible Neuroimaging Data Processing Framework in Python. *Frontiers in Neuroinformatics*, *5*. https://doi.org/10.3389/fninf.2011.00013

Harris, P. A., Taylor, R., Thielke, R., Payne, J., Gonzalez, N., & Conde, J. G. (2009). Research electronic data capture (REDCap)—A metadata-driven methodology and workflow process for providing translational research informatics support. *Journal of Biomedical Informatics*, *42*(2), 377–381. https://doi.org/10.1016/j.jbi.2008.08.010

Hennig, J., Boehm, I., Zwosta, K., King, J. A., Geisler, D., Ruge, H., Seidel, M., Bernardoni, F., Hellerhoff, I., Doose, A., Pauligk, S., Leschzinski, H., Roessner, V., Wolfensteller, U., & Ehrlich, S. (2025). Avoidance habit learning in adolescents and young women with anorexia nervosa: An fMRI study. *Journal of Child Psychology and Psychiatry*, jcpp.70019. https://doi.org/10.1111/jcpp.70019

JASP Team. (2024). *JASP* (Version 0.19.1) [Computer software]. https://jasp-stats.org/

King, J. A., Geisler, D., Bernardoni, F., Ritschel, F., Böhm, I., Seidel, M., Mennigen, E., Ripke, S., Smolka, M. N., Roessner, V., & Ehrlich, S. (2016). Altered Neural Efficiency of Decision Making During Temporal Reward Discounting in Anorexia Nervosa. *Journal of the American Academy of Child & Adolescent Psychiatry*, *55*(11), 972–979. https://doi.org/10.1016/j.jaac.2016.08.005

Roche, A. (2011). A Four-Dimensional Registration Algorithm With Application to Joint Correction of Motion and Slice Timing in fMRI. *IEEE Transactions on Medical Imaging*, *30*(8), 1546–1554. https://doi.org/10.1109/TMI.2011.2131152

Rolls, E. T., Huang, C.-C., Lin, C.-P., Feng, J., & Joliot, M. (2020). Automated anatomical labelling atlas 3. *NeuroImage*, *206*, 116189. https://doi.org/10.1016/j.neuroimage.2019.116189

Seidel, M., Wronski, M.-L., Bernardoni, F., Hennig, J., Poller, N., Locke, A., Stender, E., Heckel, S., Roessner, V., & Ehrlich, S. (2024). A longitudinal study on the change of eating disorder-specific and nonspecific habits during weight rehabilitation in anorexia nervosa. *International Journal of Clinical and Health Psychology*, *24*(4), 100522. https://doi.org/10.1016/j.ijchp.2024.100522

Sheehan, D. V., Lecrubier, Y., Sheehan, K. H., Amorim, P., Janavs, J., Weiller, E., Hergueta, T., Baker, R., & Dunbar, G. C. (1998). The Mini-International Neuropsychiatric Interview (M.I.N.I.): The development and validation of a structured diagnostic psychiatric interview for DSM-IV and ICD-10. *The Journal of Clinical Psychiatry*, *59 Suppl 20*, 22-33;quiz 34-57.

Taylor, P. A., Reynolds, R. C., Calhoun, V., Gonzalez-Castillo, J., Handwerker, D. A., Bandettini, P. A., Mejia, A. F., & Chen, G. (2023). Highlight results, don’t hide them: Enhance interpretation, reduce biases and improve reproducibility. *NeuroImage*, *274*, 120138. https://doi.org/10.1016/j.neuroimage.2023.120138

Van Den Bergh, D., Wagenmakers, E.-J., & Aust, F. (2023). Bayesian Repeated-Measures Analysis of Variance: An Updated Methodology Implemented in JASP. *Advances in Methods and Practices in Psychological Science*, *6*(2), 25152459231168024. https://doi.org/10.1177/25152459231168024

von Aster, M., Neubauer, A., & Horn, R. (2006). Wechsler Intelligenztest für Erwachsene (WIE). In *Deutschsprachige Bearbeitung und Adaptation des WAIS-III von David Wechsler*. Harcourt Test Services.

Wagenmakers, E.-J., Love, J., Marsman, M., Jamil, T., Ly, A., Verhagen, J., Selker, R., Gronau, Q. F., Dropmann, D., Boutin, B., Meerhoff, F., Knight, P., Raj, A., Van Kesteren, E.-J., Van Doorn, J., Šmíra, M., Epskamp, S., Etz, A., Matzke, D., … Morey, R. D. (2018). Bayesian inference for psychology. Part II: Example applications with JASP. *Psychonomic Bulletin & Review*, *25*(1), 58–76. https://doi.org/10.3758/s13423-017-1323-7

Zwosta, K., Ruge, H., Goschke, T., & Wolfensteller, U. (2018). Habit strength is predicted by activity dynamics in goal-directed brain systems during training. *NeuroImage*, *165*, 125–137. https://doi.org/10.1016/j.neuroimage.2017.09.062
